# Ramping Up Antimicrobial Peptides Against Severe Acute Respiratory Syndrome Coronavirus-2

**DOI:** 10.3389/fmolb.2021.620806

**Published:** 2021-06-21

**Authors:** Santosh K. Ghosh, Aaron Weinberg

**Affiliations:** Department of Biological Sciences, Case Western Reserve University, Cleveland, OH, United States

**Keywords:** antimicrobial peptides (AMPs), coronavirus, COVID-19, defensins, LL-37, MSCs, vitamin D3

## Abstract

Human-derived antimicrobial peptides (AMPs), such as defensins and cathelicidin LL-37, are members of the innate immune system and play a crucial role in early pulmonary defense against viruses. These AMPs achieve viral inhibition through a variety of mechanisms including, but not limited to, direct binding to virions, binding to and modulating host cell-surface receptors, blocking viral replication, and aggregation of viral particles and indirectly by functioning as chemokines to enhance or curb adaptive immune responses. Given the fact that we are in a pandemic of unprecedented severity and the urgent need for therapeutic options to combat severe acute respiratory syndrome coronavirus-2 (SARS-CoV-2), naturally expressed AMPs and their derivatives have the potential to combat coronavirus disease 2019 (COVID-19) and impede viral infectivity in various ways. Provided the fact that development of effective treatments is an urgent public health priority, AMPs and their derivatives are being explored as potential prophylactic and therapeutic candidates. Additionally, cell-based platforms such as human mesenchymal stem cell (hMSC) therapy are showing success in saving the lives of severely ill patients infected with SARS-CoV-2. This could be partially due to AMPs released from hMSCs that also act as immunological rheostats to modulate the host inflammatory response. This review highlights the utilization of AMPs in strategies that could be implemented as novel therapeutics, either alone or in combination with other platforms, to treat CoV-2–infected individuals.

## Introduction

The ongoing coronavirus disease 2019 (COVID-19) pandemic, the result of infection by SARS-CoV-2, continues to spread worldwide and has already claimed the lives of over three million people (John Hopkins University, 2020). This untenable situation requires the discovery of novel therapeutic approaches, alone and/or in conjunction with existing approved regimens, to impede the virus’s relentless spread. Naturally occurring cationic peptides with broad-spectrum microbicidal activity, referred to as antimicrobial peptides (AMPs), are a key component of our body’s innate immune defense against bacteria, fungi, and viruses (Brice and Diamond, 2020). Much of their antimicrobial activity is dependent upon electrostatic interactions between anionic components of microbial membranes and AMP cationic charges. Since the AMPs possess a wide range of activities in modulating the functions of various host cells as part of innate immunity, they have also been referred to in the literature as host defense peptides (HDPs) and cationic host defense peptides (CHDPs) (Hancock et al., 2016; Mookherjee et al., 2020). AMPs are known to kill microbes through direct activity involving a variety of mechanisms, including membrane permeation, disruption of electrochemical gradients, and inhibition of metabolic processes (Brogden, 2005; Prasad et al., 2019). Furthermore, these peptides interact with multiple receptors on host cells, such as toll-like receptors (TLRs) and chemokine receptors, as well as inflammasomes and members of the host’s complement system, thereby providing a bridge between innate and adaptive immunity (Hancock et al., 2016; Prasad et al., 2019; Mookherjee et al., 2020). Although more classes of AMPs occur in humans, the α- and β-defensins and cathelicidin LL-37 have been studied the most (Doss et al., 2010; Ahmed et al., 2019a; Brice and Diamond, 2020). Herein, we review how these AMPs act as antiviral agents and discuss how they may also be exploited to address SARS-CoV-2.

### An Overview of Antimicrobial Peptides: Defensins and LL-37

Defensins are a family of small (3–5 kDa), β-sheeted, cysteine-rich, cationic, and amphipathic peptides, which belong to either the α, β, or θ subfamily. α-Defensins are found in lysosomal compartments of neutrophils and macrophages, as well as in Paneth cells within the crypts of the small intestines. There are four human neutrophil α-defensins referred to as human neutrophil peptides (HNPs) 1, 2, 3, and 4. All four play an important role in killing phagocytosed microbes by professional phagocytes (Soehnlein et al., 2008; Nordenfelt and Tapper, 2011). Enteric α-defensins are referred to as human defensins (HDs) 5 and 6, where HD5 plays an important role in maintaining microbial homeostasis of the gut microbiota, while HD6 forms nets to entrap gastrointestinal pathogens within the lumen and prevent them from invading gut tissue (Chairatana and Nolan, 2017). Human β-defensins (hBDs)-1, -2, -3, and -4 are expressed in epithelial cells of various mucosal sites and participate in the mucosal innate immune defense against microbial colonization and invasion (Suarez-Carmona et al., 2015). Theta (θ)-defensins are produced by old world monkeys and orangutans but not humans. Human θ-defensin genes contain a premature stop codon that prevents effective translation (Lehrer et al., 2012). Synthetic θ-defensins with sequences that correspond to those that are encoded within the human pseudogenes are called retrocyclins (Lehrer et al., 2012). The antiviral activity of defensins was originally attributed to their lipid perturbation activity, as disruption of viral–host protein receptor interaction by lipid perturbation of the viral membrane leads to the inhibition of receptor binding so that fusion of the enveloped virus to host cells is prevented (Wilson et al., 2013). However, the observation that several classes of non-enveloped viruses are also sensitive to defensins led to the discovery of additional defensin-related antiviral mechanisms (Wilson et al., 2013; Park et al., 2018; Brice and Diamond, 2020). These include extracellular viral aggregation, blocking uncoating of the virus, and preventing nuclear import (Wilson et al., 2013). Additional AMP activities against enveloped viruses include blocking virus binding to host receptors, receptor downregulation, inhibition of viral fusion with the host membrane, blocking reverse transcription, modulating cell signaling, and blocking gene expression (Quiñones-Mateu et al., 2003; Furci et al., 2007; Kota et al., 2008; Wilson et al., 2013; Park et al., 2018).

The only member of the cathelin family of AMPs in humans is referred to as the human cationic antimicrobial peptide (hCAP) (Zanetti et al., 1995). The “cathelin” name refers to the conserved domain in the pro-peptide that is part of the inactive precursor, and the entire protein has a molecular weight of 18 kDa; hence, it is also referred to as hCAP18. The active C-terminal–associated 37 amino acid of hCAP18, starting with double leucines, is referred to as LL-37 (Xhindoli et al., 2016). LL-37 possesses direct microbicidal activity against bacteria, fungi, and multiple enveloped and non-enveloped viruses (Doss et al., 2010; Barlow et al., 2011; Xhindoli et al., 2016; Brice and Diamond, 2020; Chessa et al., 2020) including several respiratory viruses (Currie et al., 2013; Currie et al., 2016; Harcourt et al., 2016; Sousa et al., 2017). LL-37 also acts to modulate immune responses and functions in concert with TLRs to communicate an imminent threat to the immune system (Barlow et al., 2011; Ahmed et al., 2019b).

### Antimicrobial Peptides Against Respiratory Viruses: Modes of Action

Bacteria and viruses are recurring causative agents of pulmonary diseases in humans, with respiratory viruses playing a disproportionately higher etiological role (Denny, 1995; Leung, 2021). They contribute to a significant impact on morbidity, mortality, and economics worldwide, as documented and chronicled by the World Health Organization (GBD 2017 Influenza Collaborators, 2019). The most common respiratory viruses are influenza A virus (IAV), respiratory syncytial virus (RSV), parainfluenza virus (PIV), metapneumovirus (MPV), human rhinovirus (HRV), human adenovirus (HAdV), bocavirus (BoV), and coronavirus (CoV) (Boncristiani et al., 2009; Weston et al., 2019).

Defensins and LL-37 have demonstrated antiviral activities against a variety of these viruses (summarized in Table 1) including coronaviruses (see below). The potency against any particular virus varies for different peptides; for example, the potency of LL-37 for IAV inhibition has been found to be similar to that of HNPs, greater than that of human β-defensins, but less than that of retrocyclins (Tripathi et al., 2013; Doss et al., 2009). The mode of action of AMPs for these viruses (Table 1) may also differ between types (α- or β-) and subtypes (HNP1–4, HD5, HD6; hBD1–4) of defensins, and between defensins and LL-37 (Park et al., 2018; Brice and Diamond, 2020; Chessa et al., 2020); however, the most common antiviral mode of action, *in vitro*, is the capacity of AMPs to destabilize the viral envelope on contact, damaging the virions and inhibiting infectivity (Currie et al., 2013; Tripathi et al., 2014; Currie et al., 2016; Harcourt et al., 2016).

**TABLE 1:** Modes of action of defensins and LL-37 against respiratory viruses.

Viruses	AMPs	Modes of action
IAV	HNP1	•Aggregates IAV and enhances neutrophil-mediated clearance [Hartshorn et al. (2006), Tecle et al. (2007), Doss et al. (2009)]
		•Inhibits IAV replication through the inhibition of protein kinase C (PKC) in infected cells [Salvatore et al. (2007)]
	HD5	•Aggregates IAV and enhances neutrophil-mediated clearance [Tecle et al. (2007); Doss et al. (2009)]
	hBD-2	
	hBD-3	•Blocks viral fusion by creating a protective barrier of immobilized surface glycoproteins [Leikina et al. (2005)]
	Retrocyclin	•Aggregates IAV [Doss et al. (2009)]
	LL-37	•Causes disruption of viral membranes [Tripathi et al. (2013)]
RSV	hBD-2	•Blocks viral entry by destabilizing/disintegrating the viral envelope [Kota et al. (2008)]
	LL-37	•Inhibits new infectious particles and diminishes the spread of infection [Currie et al. (2013)]
		•Directly damages the viral envelope and disrupts viral particles [Currie et al. (2016)]
HAdV	HD5	•Blocks viral-mediated endosomal penetration [Smith and Nemerow (2008)]
HRV	LL-37	•Promotes reduction of the metabolic activity of infected cells [Sousa et al. (2017)]

### Antimicrobial Peptides and Their Derivatives: Activities Against Coronaviruses

All coronaviruses, including SARS-CoV-2, contain four structural proteins, known as the S (spike), E (envelope), M (membrane), and N (nucleocapsid) proteins (Siu et al., 2008; Yoshimoto, 2020). Among these, the most pertinent to studies of AMP-related activities against coronaviruses is the S protein. It comprises two functional subunits, i.e., S1 and S2 (Tortorici and Veesler, 2019), where S1 binds to host cell receptor angiotensin converting enzyme 2 (ACE2), followed by fusion of the viral and cellular membranes *via* S2 (Hoffmann et al., 2020). β-Defensins and LL-37 naturally serve as antimicrobials at vulnerable mucosal sites of our body and are primed to function as “disruptors” of viral attachment, entry, and infection. With demonstrated diverse mechanisms of action against multiple different viruses including respiratory viruses, these AMPs are obvious candidates to explore as possible anti-SARS-CoV-2 agents. In addition to their central role in innate immunity, it is becoming clear that AMPs can modulate the adaptive immune response as well (Scott et al., 2002; Diamond et al., 2009; Semple and Dorin, 2012; Koeninger et al., 2020; Liang and Diana, 2020), and several studies have demonstrated adjuvant activities of AMPs *in vivo* (Tani et al., 2000; Biragyn et al., 2002; Brogden et al., 2003; Kohlgraf et al., 2010; Mei et al., 2012). The section below will focus on evidence that AMPs and their smaller peptide derivatives have demonstrated *in vitro* and *in vivo* anti-coronaviral activity and set the stage for their consideration as antagonists of SARS-CoV-2.

#### Direct and Indirect Actions of Antimicrobial Peptides

An *in silico* study conducted by Mustafa et al. (2019) showed that a short peptide, referred to as P9, derived from mouse β-defensin 4 (an ortholog of hBD-2 (Jia et al., 2000)) binds to the type I transmembrane glycoprotein S2 domain of MERS-CoV. An *in vivo* study by Zhao H. et al. (2016) demonstrated that the P9 peptide has broad-spectrum antiviral activity against different subtypes of IAV, as well as two coronaviruses, SARS-CoV and MERS-CoV. The half-maximal inhibitory concentration (IC_50_) values of P9 against both SARS-CoV and MERS-CoV were ∼5 μg/ml. One dose of P9 for prophylaxis and five doses of P9 for therapy significantly inhibited SARS-CoV infection in mouse lungs, and the antiviral activity of P9 was attributed to its binding to the viral S2 protein, confirming the *in silico* work of Mustafa et al. (2019). Moreover, the abundance of basic amino acids in P9’s composition prevented acidification in endosomes and inhibited viral RNA release. Recently, Zhao et al. (2020) demonstrated that another short peptide, P9R, which has more net positive charge (+5.6 compared to +4.7 of P9), inhibits not only MERS-CoV and SARS-CoV but also SARS-CoV-2 (IC_50_ values: 2.2, 2.4, and 0.9 μg/ml, respectively). P9R was also shown to significantly inhibit SARS-CoV-2 replication when Vero E6 cells were infected with SARS-CoV-2, 6 and 24 h prior to the addition of P9R (Zhao et al., 2020).

Rhesus θ-defensin 1 (RTD-1) (Lehrer et al., 2012) showed efficacy as a prophylactic antivirus in a mouse model of severe SARS-CoV–induced lung disease (Wohlford-Lenane et al., 2009). BALB/c mice exposed to a mouse-adapted strain of SARS-CoV demonstrated 100% survival and reduction in lung pathology when treated with two intranasal doses of RTD-1, while mortality in untreated mice was ∼75%. RTD-1–treated SARS-CoV–infected mice displayed reductions in levels of RANTES, IL-1α, IL-1β, IL-6, IL-12, and monocyte chemoattractant protein 1α (MCP-1α), compared to untreated SARS-CoV–infected mice (Wohlford-Lenane et al., 2009).

#### Antimicrobial Peptides as Viral Binding Inhibitors That Can Block CoV-2 Entry

Multiple therapeutic approaches are currently being considered in attempts to block the CoV-2 S:ACE2 interaction to avoid viral fusion with the cell’s membrane and entry into the cell (Whisenant and Burgess, 2020). The intestinal α-defensin HD5, released from Paneth cells in the crypts of the small intestine, was recently found to bind ACE2 in a study conducted by Wang C. et al. (2020). The authors reported that HD5 bound to several ACE2 sites crucial for binding to the S protein-receptor-binding domain (S-RBD) of CoV-2 and demonstrated proof of principle by showing that HD5 blocked S protein–expressing pseudovirions from entering ACE2-expressing enterocytes. The authors surmised that this could represent innate protection of intestinal cells against CoV-2 infection.

Recent *in silico* molecular docking studies predicted strong binding interactions of LL-37 (Lokhande et al., 2020) and hBD-2 (Zhang L et al., 2021) with the receptor-binding domain (RBD) of SARS-CoV-2, suggesting an RBD blocking potential for these two peptides. Biophysical assays using bio-layer interferometry (BLI) and microscale thermophoresis (MST) supported the *in silico* findings for LL-37 (Wang C. et al., 2021) and hBD-2 (Zhang L. et al., 2021), respectively. Additionally, biochemical studies with hBD-2 showed that it inhibited the RBD from binding ACE2 and prevented S protein–expressing pseudovirions from infecting ACE2-expressing human cells (Zhang L. et al., 2021).

Interestingly, LL-37 has been found to suppress S pseudovirion infection in a dose-dependent manner with an IC_50_ value of 1.05 μM (Wang C. et al., 2021). By using a clever *in vivo* model that incorporated ACE2-expressing adenovirions, either with or without S protein–expressing pseudovirions, Wang J. et al. (2021) were able to show that intranasal administration of LL-37 protected mice from pulmonary infection (Wang C. et al., 2021). This is the first demonstration of a natural AMP that can inhibit CoV-2 entry *via* a dual mechanism.

It is important to note that while the focus on blocking SARS-CoV-2 entry into vulnerable cells is *via* ACE2, a new discovery highlights that neuropilin-1 (NRP1), a receptor involved in multiple physiological processes and expressed on many cell types (Roy et al., 2017), is being utilized by the virus to facilitate entry and infection (Cantuti-Castelvetri et al., 2020; Daly et al., 2020). Teesalu et al. (2009) showed that a peptide with an internal R/KXXR/K motif can bind to NRP1 (Teesalu et al., 2009). Interestingly, both hBD-2 and -3 have these motifs near their respective C-terminal ends (KCCK for hBD-2 and KCCR for hBD-3) (Yamaguchi et al., 2002). Therefore, we cannot rule out the possibility of defensins binding to NRP1. However, time will tell if defensins are effective in blocking viral entry *via* NRP1.

#### Antimicrobial Peptides as Adjuvants

hBD-2 and -3 have previously been used as adjuvants to design multi-epitope vaccines against MERS-CoV, utilizing several *in silico* methods and tools (Srivastava et al., 2018). Kim et al. (2018) found that C57BL/6 mice immunized with hBD-2 conjugated to MERS-CoV-S-RBD (hBD-2/MERS-CoV-S-RBD) had significantly higher S-RBD–specific IgG titer levels in comparison with those receiving S-RBD alone. When hBD-2/MERS-CoV-S-RBD was used to treat THP-1 monocytic cells, the expression levels of classical antiviral (IFN-β, IFN-γ, PKR, and RNaseL) and primary immune-inducing molecules (NOD2, TNF-α, IL-1β, and IL-6) were enhanced compared to expression levels after treatment with only S-RBD. The receptor-binding inhibition assay on the MERS-CoV–susceptible Vero E6 cell line using sera obtained from mice immunized with PBS, S-RBD, or hBD-2/MERS-CoV-S-RBD showed that sera from hBD-2–conjugated S-RBD–inoculated mice almost completely inhibited S-RBD binding to cell surfaces compared with sera from mice immunized with S-RBD alone. hBD-2–conjugated S-RBD was also superior to unconjugated S-RBD in inducing neutralizing antibodies against MERS-CoV infection. A more recent study showed that immunization with S RBD-hBD-2 alleviated progressive pulmonary fibrosis in the lungs of MERS-CoV–infected mice and suppressed endoplasmic reticulum stress signaling activation upon viral infection (Kim et al., 2020).

A multi-epitope vaccine against SARS-CoV-2 using hBD-3 conjugated to B-cell, helper T-lymphocyte (HTL), and cytotoxic T-lymphocyte (CTL) epitopes was designed using *in silico* structural biology and immunoinformatic approaches (Whisenant and Burgess, 2020). When tested using the C-ImmSim server (Rapin et al., 2010), which simulates the natural immune outcome, the multi-epitope vaccine generated a robust response by B-cells, T helper cells, cytotoxic T cells, and IgG (Ojha et al., 2020). While *in silico* findings suggest that this vaccine (Ojha et al., 2020) and others using hBD-3 as the adjuvant (Dong et al., 2020; Yazdani et al., 2020) are promising potential therapeutic approaches against COVID-19, *in vivo* studies need to be conducted to prove their effectiveness against COVID-19.

### Vitamin D Deficiency and COVID-19: A Possible Link With Antimicrobial Peptides

The genes encoding the β-defensins and LL-37 contain consensus vitamin D_3_ (Vit D_3_) response elements (VREs) (Wang et al., 2004; McMahon et al., 2011; Aguilar-Jimenez et al., 2013), and it is well established that Vit D_3_ and its metabolite 1,25-dihydroxy-vitamin D_3_ regulate the AMPs’ expressions (Wang et al., 2004; Adams et al., 2009). Vit D_3_ deficiencies have been associated with an increase in inflammatory cytokines and significant susceptibility to pneumonia and upper respiratory tract infections (Weir et al., 2020); both are common outcomes in severely ill COVID-19 patients (Chen et al., 2020; Ding et al., 2020; Leisman et al., 2020). Indeed, several studies suggest that Vit D_3_ may have beneficial properties against SARS-CoV-2, as individuals deficient in Vit D_3_ appear to be more susceptible to contracting the virus than those whose levels are normal (Arvinte et al., 2020; Hernández et al., 2020; Kaufman et al., 2020; Radujkovic et al., 2020). Additionally, reduced levels of VREs (important in AMP induction (McMahon et al., 2011; Aguilar-Jimenez et al., 2013)) in cells isolated from bronchoalveolar lavage were found in patients infected with CoV-2 than healthy subjects (George et al., 2020). Moreover, the fact that LL-37 (Zhang et al., 2020) and hBD-2 (Zhang L. et al., 2021), both regulated through Vit D_3_, were able to block S protein–expressing pseudovirions from infecting vulnerable human cells supports the notion that healthy levels of Vit D_3_ may be important in reducing the risk of acquiring SARS-CoV-2 infection. However, further studies on the direct correlation between Vit D_3_ and AMP levels in relation to susceptibility to CoV-2 acquisition are required.

### Mesenchymal Stem Cells and COVID-19: Plausible Role for Antimicrobial Peptides

Among several approaches repurposed to treat COVID-19 patients, human mesenchymal stem cell (hMSC) therapy has recently been reported to contribute to the recovery of severely ill CoV-2–infected patients (Moll et al., 2020; Tsuchiya et al., 2020). With a 100% survival rate using hMSCs in compassionate use programs to treat severely ill COVID-19 patients (Israeli COVID-19 treatment shows 100% survival rate-preliminary data, Jerusalem Post), several biotech companies and universities are conducting clinical trials to evaluate their respective cell therapy platforms. The mechanisms behind hMSC therapeutic benefits are presently a “black box,” although some evidence points to the ability of these cells to modulate severe inflammation by secreting several beneficial agents (Iannaccone et al., 2020; Rajarshi et al., 2020; Tsuchiya et al., 2020). Supernatants from activated hMSCs kill microbes associated with cystic fibrosis, and cystic fibrotic mice, which otherwise would succumb to microbial infections, survive these challenges by injection of hMSCs (Krasnodembskaya et al., 2010; Sutton et al., 2016; Alcayaga-Miranda et al., 2017; Chow et al., 2020). These favorable outcomes can be partly attributed to hMSC-released AMPs, such as defensins and LL-37 (Krasnodembskaya et al., 2010; Sutton et al., 2016), which are found in bronchial alveolar lavage (Ghosh et al., 2007; Golec et al., 2012). Do AMPs actually contribute to cessation of inflammation currently attributed to hMSC activity, in addition to directly inhibiting viral infection or not? With growing evidence that defensins and LL-37 have anti-inflammatory properties (Choi et al., 2012; Mansour et al., 2014; Brook et al., 2016), along with their diverse strategies to directly attack viruses, it will be a tall order to dissect out the inflammomodulatory role that hMSC-related AMPs play in cessation of the cytokine storm that afflicts severely ill CoV-2–infected patients. Finding the right balance of anti-inflammatory vs. pro-inflammatory activities, so that we do not inadvertently exacerbate an already inflamed situation, will require in depth testing of each AMP. These could include determining conformational status, identifying the distinct isoform and amino acid motifs important for each modulatory activity, and designing novel synthetic derivatives from modifications of natural AMPs to then test in both *in vitro* and *in vivo* models. This has been an approach espoused by Robert Hancock’s group, which they address in a review article (Haney et al., 2019). Moreover, hMSC-related AMPs, which could include additional yet-to-be–discovered peptides, could be interacting in synergy with other beneficial agents secreted by hMSCs, such as exosomal agents that limit immune thrombosis, increase fibrinolytic activity, re-stabilize endothelial integrity, reduce lymphocyte trafficking, and promote recruitment of M2 macrophages and regulatory T cells (Gomzikova et al., 2019; Jamshidi et al., 2021; Moradinasab et al., 2021; Su et al., 2021).

## Conclusion and Perspectives

The saying that “*desperate diseases call for desperate treatments*” (attributed to Hippocrates) cannot be more appropriate during these trying times when a worldwide pandemic is wreaking havoc on mankind. It is clear that naturally occurring AMPs, such as defensins and LL-37, possess favorable properties that make them prime candidates for novel anti-COVID-19 therapeutics. They can act directly and indirectly against coronaviruses (Wohlford-Lenane et al., 2009; Zhao G. et al., 2016; Zhang et al., 2020; Zhao et al., 2020), and they are especially effective at blocking viral entry into vulnerable cells (Wang R.et al., 2020), small peptide derivatives (Zhao et al., 2020), and non-peptide mimetics (Bakovic et al., 2020). Of these, AMPs are emerging as promising drug candidates, and they can be used as adjuvants (Kim et al., 2018; Dong et al., 2020; Ojha et al., 2020) in vaccines targeting coronaviruses. Additionally, the importance of Vit D_3_ in protection against SARS-CoV-2 acquisition and the repurposed use of hMSCs in treating severe cases of COVID-19 point to the possible benefits of AMP protection. Importantly, designing small peptides from human AMPs has multiple advantages. They possess safety, i.e., limiting the need for phase 1 studies, they are highly specific, they could be designed to resist peptidase biodegradability, they are not expensive to produce, and they could be administered easily (Greber and Dawgul, 2017; Di et al., 2020; Luong et al., 2020). Currently, more than 30 AMPs including LL-37 are in clinical and preclinical trials for their potential applications against various infectious diseases (Koo and Seo, 2019).

Several conventional therapeutic drugs, including but not limited to antimalarial drugs, protease inhibitors, renin–angiotensin system (RAS) inhibitors, inhibitors of the RNA-dependent RNA polymerase, and immune suppressants, are being repurposed for the treatment of COVID-19 (Khavinson et al., 2020; Spaccarotella et al., 2021). However, peptide-based therapeutic drugs including AMPs are sometimes a better choice than conventional drugs due to their higher efficacy, lower molecular weight, and lower toxicity and side effects (Castel et al., 2011). Among peptide-based therapeutic drugs, AMP-related small peptide derivatives (Zhao et al., 2020) and non-peptide mimetics (Bakovic et al., 2020) are emerging as promising drug candidates. Several potential SARS-CoV-2 entry inhibitor peptides, and strategies used to design those peptides targeting the ACE2 receptor or the viral spike protein and its activating proteases, have been outlined in a recent review by Schütz et al. (2020). Additionally, peptoid mimics (sequence-specific *N*-substituted glycine oligomers) of AMPs and an antimicrobial DP7 peptide (VQWRIRVAVIRK) were recently shown to have anti-CoV-2 activity (Zhang R. et al., 2021; Diamond et al., 2021). Moreover, a synthetic mimetic of defensins, *Brilacidin*, has been shown to potently inhibit CoV-2 in an ACE2-positive human lung cell line (Bakovic et al., 2021) and recently received approval by the Federal Drug Administration (FDA) to start a phase 2 clinical trial in COVID-19 patients (ClinicalTrials.gov; Identifier: NCT04784897).

Since AMPs are highly sensitive to environmental conditions, such as pH and ionic strength, which often leads to discrepancies between *in vitro* and *in vivo* results (Mahlapuu et al., 2016), improving them as viable therapeutics is being addressed through peptide mimetics (Pachón-Ibáñez et al., 2017; Mookherjee et al., 2020). These are being engineered, using the AMP backbone, to increase cationicity and amphipathicity, when needed, with minimal cytotoxicity; for example, a number of shorter LL-37 variants have been generated to improve the antimicrobial activity and reduce the toxicity (Tripathi et al., 2015; Pachón-Ibáñez et al., 2017). Low metabolic stability of AMPs, an additional challenge for therapeutics, is being addressed by modifying the peptide backbone through incorporation of d-amino acids, end-tagging by hydrophobic amino acid stretches, and blocking N- and/or C-terminal ends of the peptide by N-acetylation or C-amidation (Zhao Y. et al., 2016; Håkansson et al., 2019; Mahlapuu et al., 2020).

**FIGURE 1:**
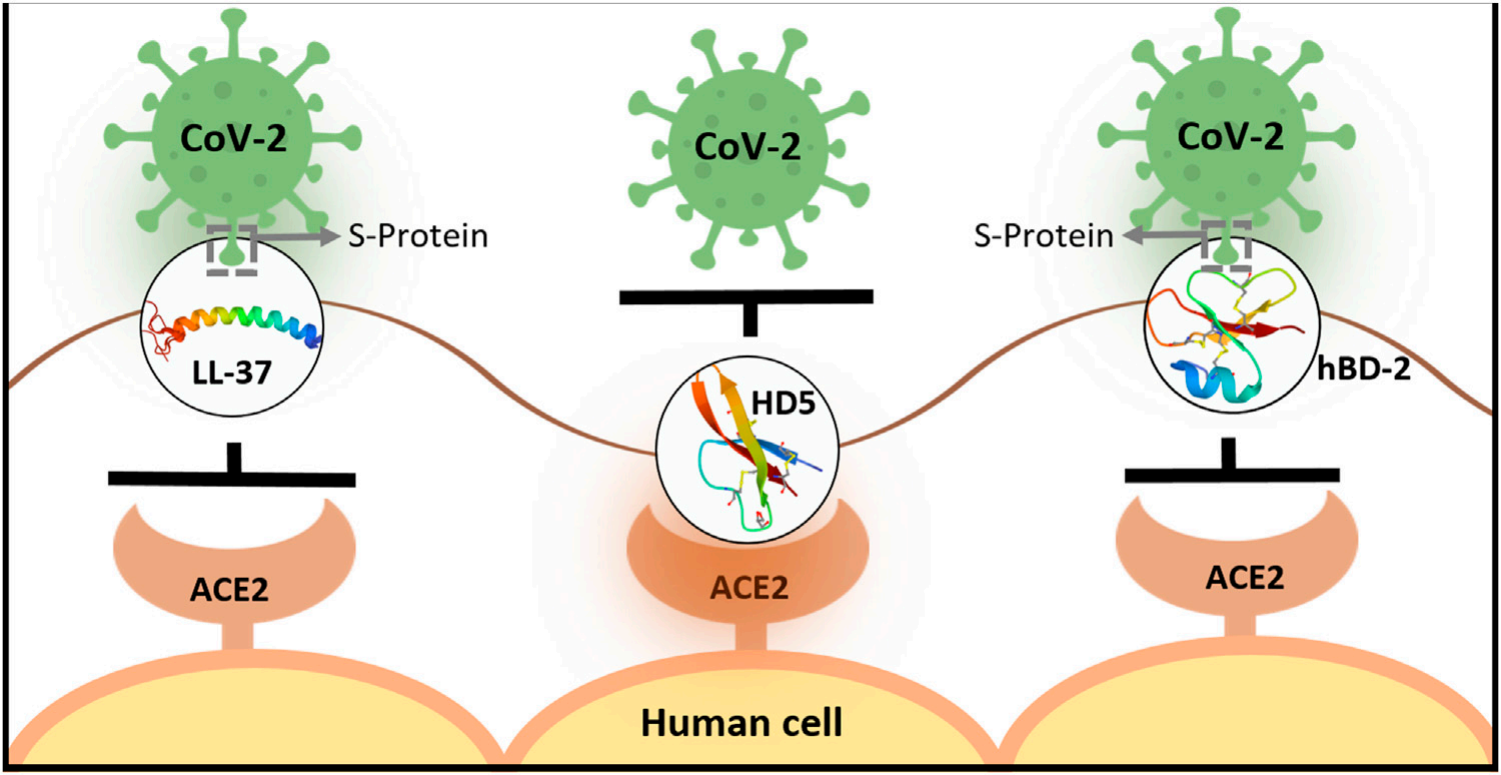
Schematic representation showing differential binding of human AMPs to SARS-CoV-2 S protein or ACE2 on cell surfaces. LL-37 and hBD-2 bind to SARS-CoV-2 S protein and inhibit its binding to ACE2 on the cell surface prior to entry (Lokhande et al., 2020; Roth et al., 2020; Wang C. et al., 2021; Zhang L. et al., 2021). In contrast, HD-5 binds to ACE2 to inhibit viral entry (Wang et al., 2020a) [LL-37 has also been shown to bind ACE2, however, with lower affinity than S-RBD (Wang C. et al., 2021)].

The nasal cavity and nasopharynx contain some of the highest viral loads in the body, and viral load levels are similar in symptomatic and asymptomatic individuals. The so-called “silent spreaders” may involuntarily contribute to the exponential growth of disease, as nasal secretions contain spreadable virus (Higgins et al., 2020) but lack endogenous expression of some of the AMPs [e.g., hBD-2 (Guaní-Guerra et al., 2011; Bouloukaki et al., 2011)]. Additionally, nasopharyngeal swab samples have revealed that CoV-2–infected patients have lower mRNA levels of several defensins when compared to uninfected subjects (Idris et al., 2020). To “ramp up” AMP levels, AMPs and/or their derivatives could be administered intranasally and/or intraorally as prophylactic aerosols, in early stages of infection when telltale symptoms begin to appear and in combinatorial therapeutic approaches for more severe situations. A prophylactic strategy has been proposed by Park et al. (2018), when natural endogenous levels of constitutive or viral-induced defensins provide a limited level of defense against infecting viruses, especially with high viral loads. AMP-based therapy has additional benefits in the context of COVID-19, as different AMPs have affinities for different CoV-2 targets; for example, LL-37 (Lokhande et al., 2020; Roth et al., 2020) and hBD-2 (Zhang L. et al., 2021) bind to SARS-CoV-2 S-RBD, whereas HD5 binds to ACE2 (Wang C. et al., 2020) but not SARS-CoV-2 S-RBD [summarized in Figure 1]. Therefore, combining different AMPs that bind different targets associated with CoV-2 entry may turn out to be more beneficial than using only one AMP. Moreover, if mutations in the S-RBD (Wang et al., 2020b) preclude using a specific AMP, other AMPs targeting ACE2 could continue to block viral entry. Additionally, since AMPs and their small peptide derivatives lack immunogenicity and demonstrate low levels of toxicity (Otte et al., 2008; Warnke et al., 2013; Leelakanok et al., 2015), they are ideal candidates for both prophylactic and therapeutic approaches in dealing with SARS-CoV-2 dispersion.

We believe AMP utilization as first-line antivirals is a cogent stopgap while several vaccine candidates are being tested. Moreover, we do not see them diminishing in importance once vaccines are available to everyone, as vaccines do not always provide 100% protection (Goodwin et al., 2006; Ovsyannikova et al., 2017). Many people will refuse vaccination (Schwarzinger et al., 2010; Fisher et al., 2020; Pogue et al., 2020; Wang J. et al., 2021), and a significant number will fail to mount either effective neutralizing antibodies or high enough titers (Goodwin et al., 2006; Ndifon et al., 2009; Ovsyannikova et al., 2017). Additionally, within a few months since its appearance, CoV-2 has already developed a substantial number of mutations in the receptor-binding motif (RBM) of the RBD (Wang et al., 2020b; Abdool Karim and de Oliveira, 2021; Greaney et al., 2021). Though most mutated variants with amino acid change within the RBD were found to be less infectious, some variants have already become resistant to some neutralizing antibodies (Li et al., 2020; Hoffmann et al., 2021), warranting alternative and adjunctive approaches. There is also evidence of declining levels of neutralizing antibodies in COVID-19 patients within two to three months after recovery (Long et al., 2020; Seow et al., 2020). Given the enormity of the COVID pandemic, it is imperative to develop effective interventions capable of preventing transmission of diverse SARS-CoV-2 variants by exploiting all the possible strategies.
